# Unveiling the Impact of Morphine on Tamoxifen Metabolism in Mice *in vivo*

**DOI:** 10.3389/fonc.2020.00025

**Published:** 2020-02-21

**Authors:** Florian Gabel, Anne-Sophie Aubry, Volodya Hovhannisyan, Virginie Chavant, Ivan Weinsanto, Tando Maduna, Pascal Darbon, Yannick Goumon

**Affiliations:** ^1^CNRS UPR3212, Institut des Neurosciences Cellulaires et Intégratives, Centre National de la Recherche Scientifique, University of Strasbourg, Strasbourg, France; ^2^Mass Spectrometry Facilities of the CNRS UPR3212, Institut des Neurosciences Cellulaires et Intégratives, Centre National de la Recherche Scientifique, Strasbourg, France

**Keywords:** Tamoxifen, 4OH-tamoxifen, endoxifen, Morphine, metabolism, CYP, UDP-glucuronosyltransferase, drug-drug interactions

## Abstract

**Background:** Tamoxifen is used to treat breast cancer and cancer recurrences. After administration, tamoxifen is converted into two more potent antitumor compounds, 4OH-tamoxifen and endoxifen by the CYP3A4/5 and 2D6 enzymes in human. These active compounds are inactivated by the same UDP-glucuronosyltransferase isoforms as those involved in the metabolism of morphine. Importantly, cancer-associated pain can be treated with morphine, and the common metabolic pathway of morphine and tamoxifen suggests potential clinically relevant interactions.

**Methods:** Mouse liver microsomes were used to determine the impact of morphine on 4OH-tamoxifen metabolism *in vitro*. For *in vivo* experiments, female mice were first injected with tamoxifen alone and then with tamoxifen and morphine. Blood was collected, and LC-MS/MS was used to quantify tamoxifen, 4OH-tamoxifen, N-desmethyltamoxifen, endoxifen, 4OH-tamoxifen-glucuronide, and endoxifen-glucuronide.

**Results:** *In vitro*, we found increased *K*_m_ values for the production of 4OH-tamoxifen-glucuronide in the presence of morphine, suggesting an inhibitory effect on 4OH-tamoxifen glucuronidation. Conversely, *in vivo* morphine treatment decreased 4OH-tamoxifen levels in the blood while dramatically increasing the formation of inactive metabolites 4OH-tamoxifen-glucuronide and endoxifen-glucuronide.

**Conclusions:** Our findings emphasize the need for caution when extrapolating results from *in vitro* metabolic assays to *in vivo* drug metabolism interactions. Importantly, morphine strongly impacts tamoxifen metabolism in mice. It suggests that tamoxifen efficiency could be reduced when both drugs are co-administered in a clinical setting, e.g., to relieve pain in breast cancer patients. Further studies are needed to assess the potential for tamoxifen-morphine metabolic interactions in humans.

## Background

Breast cancer is the most common and deadliest cancer diagnosed in women, even though major advances in screening and treatment have been made in the last 20 years (1). In estrogen receptor (ER)-positive breast tumors, the main strategy of breast anticancer drugs is to either antagonize ER signaling or decrease estrogen synthesis to prevent cancer cell proliferation. Among those drugs, tamoxifen is a selective estrogen receptor modulator (SERM) used for decades to decrease breast cancer recurrence (2). Nowadays, tamoxifen remains one of the major treatment for breast cancer, especially in countries with limited health care resources (3).

**Figure 1:**
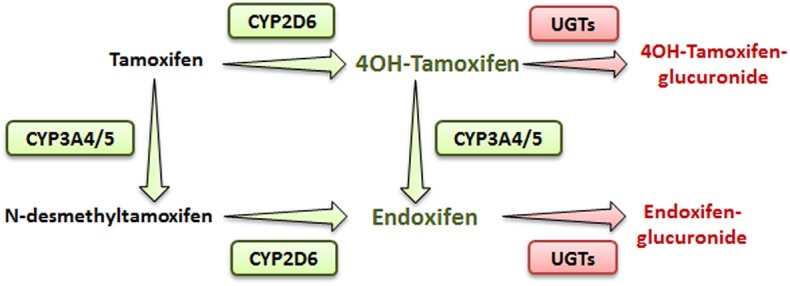
Simplified metabolic pathway of tamoxifen in humans. Compounds in green and red are the active and inactive metabolites of tamoxifen, respectively.

Tamoxifen is a pro-drug metabolized mostly in the liver by the phase I cytochrome P450 (CYP) 2D6 and 3A4/5 enzymes (4). In human, hydroxylation of tamoxifen (CYP2D6) leads to 4OH-tamoxifen that can be further processed into endoxifen (via CYP3A4/5) through N-desmethylation (Figure 1). These two major metabolites are 30- to 100-fold more potent than tamoxifen itself and are responsible for its anti-tumoral activity.

In addition, N-desmethylation of tamoxifen generates the N-desmethyltamoxifen intermediate (CYP3A4/5) that is further metabolized into endoxifen through CYP2D6-mediated hydroxylation.

Endoxifen is the major metabolite of tamoxifen in humans. Alternatively, in mice, even though CYP2D6 isoform is absent, 4OH-tamoxifen is the main anticancer product of tamoxifen, suggesting that other CYP2D isoforms, such as CYP2D22, could be involved in its metabolism (4, 5).

Phase II metabolizing enzymes including Uridine 5′-diphospho (UDP)-glucuronosyltransferases (UGT1A10, 1A4, 1A8, 2B7, and 2B15) convert active tamoxifen metabolites into inactive 4OH-tamoxifen-glucuronide and endoxifen-glucuronide (Figure 1) (6). Approximately 75% of a given dose of tamoxifen is excreted into the biliary tract as inactive glucuronides (7).

Cancer-associated pain resulting from metastases, anticancer treatment or surgery represents a major problem that is treated with analgesic drugs including morphine, codeine, and/or paracetamol (8). Morphine remains the gold standard for moderate and severe pain relief despite side effects that limit its chronic use (9). In humans, morphine acts on Mu opioid receptors (MORs) to produce analgesia. Its metabolism in the liver and brain leads mainly to the formation of morphine-3-glucuronide (M3G) (10) and morphine-6-glucuronide (M6G) (10–12). In human, morphine-glucuronidation is catalyzed by UGT2B7 and to a lower extent by a number of other UGT isoforms (UGT1A10, UGT1A1, 1A3, 1A6, 1A8, 1A9, 2A1, and UGT2B21) (9, 13, 14). However, in mice, UGT2B7 (the major enzyme involved in morphine metabolism in human) is absent but its activity is rescued by UGT2B21 and UGT2B36 (14–16).

Drug–drug interactions, resulting in either enzyme inhibition or induction, are a major limitation for the use of co-treatments (17). Usually, these drug–drug interactions are initially studied *in vitro* and then *in vivo* (18). While *in vitro* studies provide interesting results, their interpretation has proven to be complex when translated to *in vivo* drug metabolism (18).

Although anti-cancer agents share common catabolic pathways with many opiates, the impact of their co-administration on the metabolism and thus on the activity of anticancer drugs remains unexplored. These potential interactions between analgesic and anticancer drug metabolism could be used to treat more efficiently breast cancer. Therefore, as a proof of concept, we have investigated in mice whether morphine can alter tamoxifen metabolism.

## Methods

### Animals

Experiments were performed with 11- to 29-week-old female C57BL/6J mice (23 ± 4 g; Charles River, L'Arbresle, France). Animals were housed according to a 12-h light–dark cycle, at a temperature of 22°C ± 2°C and provided with food and water *ad libitum*. All procedures were performed in accordance with European directives (2010/63/EU) and were approved by the regional ethics committee and the French Ministry of Agriculture (license no. APAFIS#16827-2018092113192911 v4 to YG).

### Blood Collection

The tail of the mouse was anesthetized locally with a cutaneous application of lidocaine/prilocaïne 5% (Zentiva, Paris, France). After 5 min, a small incision was performed at the end of the tail and 10 μl of blood was collected using a calibrated capillary (Minicaps End-to-End 10 μl; Hischmann, Eberstadt Germany).

### Tamoxifen and Morphine Injections

**Figure 2:**
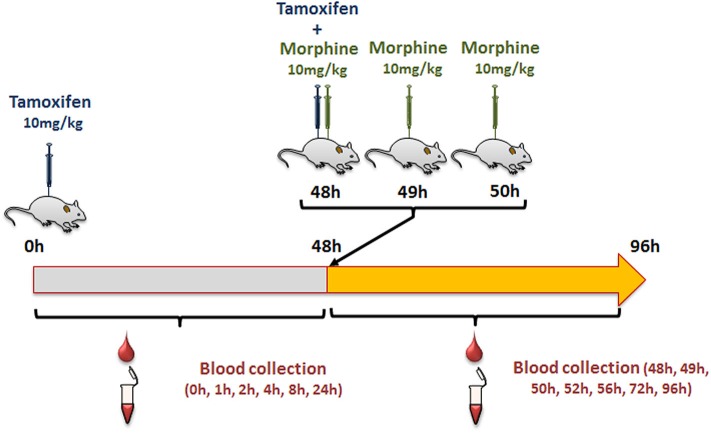
Protocol used to study tamoxifen–morphine drug–drug interactions.

Female mice were injected intraperitoneally (i.p., calibrated Hamilton syringe) with 10 mg/kg of tamoxifen (in 90% olive oil/10% ethanol, v/v; Sigma Aldrich, Lyon, France), and then with NaCl 0.9% at 0, 1, and 2 h following tamoxifen administration (Figure 2). Blood was collected by tail vein sampling (see above) just before and at 1, 2, 4, 8, 24, and 48 h after tamoxifen injection (Figure 2). A second injection of tamoxifen was then performed at 48 h and immediately followed by an injection of either 10 mg/kg of morphine–HCl (diluted in 0.9% NaCl; Francopia, Paris, France) or saline solution (0.9% NaCl only). Mice then received two additional injections of morphine or saline at 1 and 2 h after the second tamoxifen dose. Blood was collected at 1, 2, 4, 8, 24, and 48 h after the second tamoxifen injection (Figure 2).

### Sample Preparation

The blood was transferred from the capillary into a microtube containing 10 μl of heparin and frozen at −20°C. On the next day, blood was thawed and 10 μl of an internal standard (IS; see below) and 100 μl of ice-cold acetonitrile (ACN; Thermo Scientific, San Jose, USA) were added. The samples were next vortexed and centrifuged at 20,000 g during 15 min at 4°C. The supernatants were collected, dried under vacuum, and suspended in 15 μl of 50% methanol/0.1% formic acid (v/v; Sigma Aldrich) prior to LC-MS/MS analysis.

### Microsome Preparation

Liver tissues were collected from 10-week-old male C57/BL6J mice. Samples were pooled and homogenized with an Ultra Turrax instrument (Ika, Staufen, Germany) in 10 ml of extraction buffer (100 mM Na phosphate buffer, pH 7.4, 0.32 M sucrose, 1 mM EDTA, 0.1 mM DTT, protease inhibitor cOmplete Mini, EDTA-free, Roche, Basel, Switzerland). The homogenate was then sonicated (2 × 10 s, 100 W) and centrifuged for 12 min at 2,000 g (4°C). The supernatant was transferred into polycarbonate ultracentrifuge tubes (Beckman Instruments, Palo Alto, USA), completed with extraction buffer and centrifuged 40 min at 10,000 g and 4°C in a type-70 Ti Rotor (Beckman Coulter, Brea, USA). The resulting supernatant was then centrifuged for 130 min at 130,000 g (4°C), and the pellet obtained was suspended in 800 μl of storage buffer (100 mM Na phosphate buffer, pH 7.4, 0.5 mM EDTA, 0.1 mM DTT, 20% glycerol; Sigma Aldrich) and frozen. Protein concentration was determined using the Bradford method (Protein Assay, Bio-Rad, Marnes-la-Coquette, France).

### Enzymatic Activity Assay

One hundred micrograms of liver microsomes were used to perform 4OH-tamoxifen glucuronidation assays. First, increasing concentrations of 4OH-tamoxifen (10, 20, 40, 50, 60, 70, 80, 100, 125, 150, 200, 250, and 300 μM; LGC Standard, Molsheim, France) with a fixed concentration of morphine (500 μM) were dried under vacuum. Morphine was suspended in 4 mM MgCl_2_ adjusted with H_2_O, and each 4OH-tamoxifen concentration was diluted with 69 μl of the morphine-containing mix.

Microsomes were incubated for 15 min at 4°C in the presence of alamethicin (30 μg/mg of protein; Santa Cruz Biotechnology, Heidelberg, Germany) and Tris–HCl buffer (400 mM) adjusted with H_2_O. Then, 75 μl of microsome were added to each 4OH-tamoxifen concentration and tubes were equilibrated at 37°C during 5 min. The enzymatic reactions were started with the addition of 6 μl of UDPGA to a final concentration of 5 mM. Reactions were stopped 20 s later with 900 μl of cold 100% methanol. Samples were then diluted (1:5), and an IS (see below) was added to each sample. Samples were centrifuged for 15 min at 20,000 *g*, and 4°C and the supernatants were dried under vacuum and then suspended in 45 μl of 50% methanol/0.1% formic acid (v/v) prior to LC-MS/MS analysis. *K*_m_ and *V*_max_ were obtained with a Michaelis-Menten plot following a non-linear curve fit with the least-squares method (GraphPad Prism 6 software).

### LC-MS/MS Instrumentation and Analytical Conditions

Analyses were performed with a Dionex Ultimate 3000 HPLC system (Thermo Scientific) coupled with a triple quadrupole Endura mass spectrometer. Xcalibur v2.0 software was used to control the system (Thermo Electron, Villebon Sur Yvette, France). Samples were loaded onto an Accucore RP-MS column (150 × 1 mm, 2 μm, flow of 90 μl/min; Thermo Electron) heated at 40°C. Buffer A was 1% ACN/98.9% H_2_O/0.1% formic acid (v/v/v), whereas buffer B was 99.9% ACN/0.1% formic acid (v/v). The gradient used is detailed in Supplementary Table 1.

Electrospray ionization was achieved in the positive mode with the spray voltage set at 3,500 V. Nitrogen was used as the nebulizer gas, and the ionization source was heated to 250°C. Desolvation (nitrogen) sheath gas was set to 18 Arb and Aux gas was set to 7 Arb. Ion transfer tube was heated at 297°C. Q1 and Q2 resolutions were set at 0.7 FWHM, whereas collision gas (CID, argon) was set to 2 mTorr. Identification of the compounds was based on precursor ion, selective fragment ions, and retention times. Selection of the monitored transitions and optimization of collision energy and RF Lens parameters were manually determined (see Supplementary Table 1 for details). Qualification and quantification were performed in MRM mode using Quan Browser software (Thermo Scientific).

### Statistics

Statistical analysis was performed using GraphPad Prism 6 Software. Results were presented as mean values ± standard error of the mean (SEM). Groups were compared using multiple *t*-tests.

## Results

### Enzymatic Study *in vitro*

**Figure 3:**
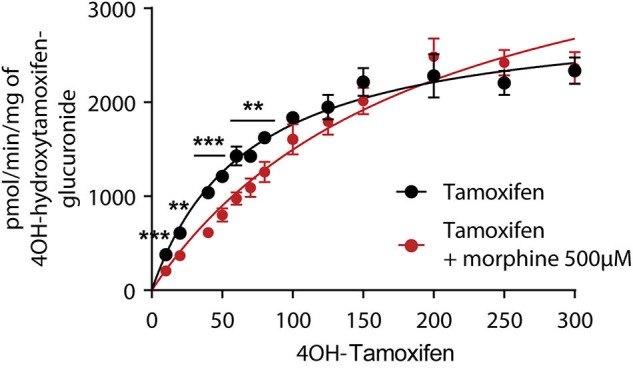
Morphine (500 μM) inhibits the formation of 4OH-tamoxifen-glucuronide *in vitro*. A *t*-test using the Holm–Sidak method was performed to compare each concentration of 4OH-tamoxifen. *n* = 7 for tamoxifen alone and *n* = 5 in the presence of morphine; **p < 0.01; ***p < 0.001. Values are means ± SEM.

As 4OH-tamoxifen is the major active metabolite of tamoxifen in mice, *in vitro* experiments were performed on mouse liver microsomes to study the impact of 500 μM of morphine on the glucuronidation of 4OH-tamoxifen. Morphine was used at 500 μM to determine the *K*_m_ of the glucuronidation of 4OH-tamoxifen as this concentration corresponds to the *K*_m_ previously determined for morphine glucuronidation in mice (12, 19). As shown in Figure 3, morphine significantly affects the production of 4OH-tamoxifen-glucuronide. Specifically, morphine significantly reduced the production of 4OH-tamoxifen-glucuronide when 10 to 50 μM and 70 μM of tamoxifen were used. *K*_m_ values for the production of 4OH-tamoxifen-glucuronide in the absence and presence of morphine, as determined by the Michaelis-Menten equation, were 68 and 98.6 μM (+45%), respectively.

These results indicate that morphine reduces 4OH-tamoxifen glucuronidation *in vitro*.

### Study of Tamoxifen Metabolism *in vivo*

**Figure 4:**
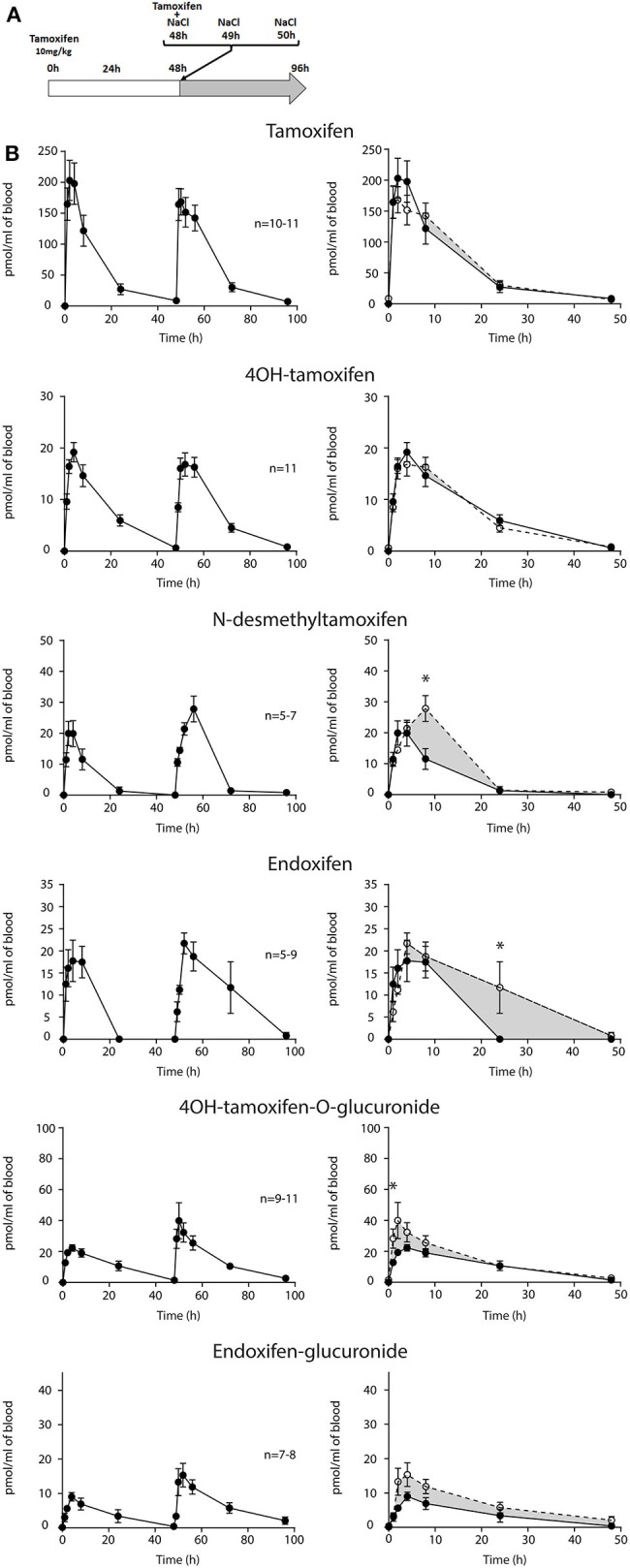
Tamoxifen metabolism is affected by a prior injection. Effect of two subsequent injections of tamoxifen (10 mg/kg i.p.) on the levels of tamoxifen and its metabolites. **(A)** Protocol. Injections of NaCl 0.9% at 0 h, 1 h, and 2 h are not represented. **(B)** Left panels, levels of tamoxifen, 4OH-tamoxifen, N-desmethyltamoxifen, endoxifen, 4OH-tamoxifen-glucuronide, and endoxifen-glucuronide during 96 h. Right panels correspond to the superimposition of the first 0–48 h (white area) and last 48–96 h (gray area). The gray area corresponds to an increase in the quantity of the corresponding molecule after the second injection (48–96 h). Multiple *t*-tests with the Holm–Sidak correction were applied. Values are means ± SEM. *p < 0.05.

**Figure 5:**
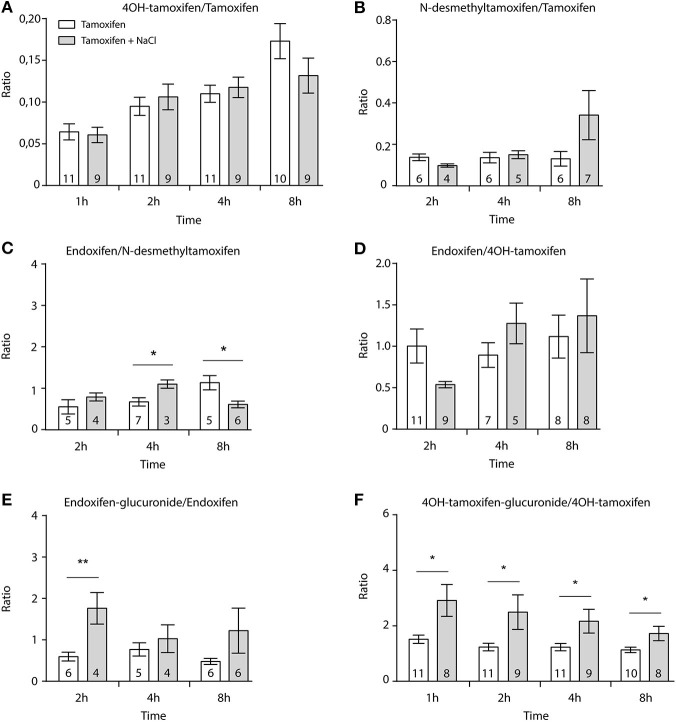
Tamoxifen potentiates its own metabolism. Ratio between metabolites and parent compounds. **(A)** 4OH-tamoxifen/tamoxifen, **(B)** N-desmethyltamoxifen/tamoxifen, **(C)** endoxifen/N-desmethyltamoxifen, **(D)** endoxifen/4OH-tamoxifen, **(E)** endoxifen-glucuronide/endoxifen, and **(F)** 4OH-tamoxifen-glucuronide/4OH-tamoxifen. *N* are indicated within columns. Values are means ± SEM. *t*-tests; *p < 0.05; **p < 0.001.

First, we determined whether multiple injections of tamoxifen would alter its own metabolism (Figures 2, 4A). Blood was collected before and 1, 2, 4, 8, 24, and 48 h after the first (Figure 4A, white part) and the second injection of tamoxifen (Figure 4A, gray part). Tamoxifen, 4OH-tamoxifen, and endoxifen-glucuronide concentrations in the blood did not vary significantly at any time point between the two tamoxifen injections (Figure 4B). In contrast, a significant increase in the concentrations of 4OH-tamoxifen-glucuronide, N-desmethyltamoxifen and endoxifen was observed. Accordingly, drug metabolic ratios (i.e., the concentration ratio of a metabolite compared to its parent molecule) were significantly altered at different time points (Figures 5A–F). The ratio of endoxifen/N-desmethyltamoxifen was significantly elevated at 4 and 8 h compared to the first injection suggesting an increase in endoxifen synthesis (Figure 5C). In a more dramatic manner, 4OH-tamoxifen glucuronidation was increased by 1.5- to 2-fold at all time points compared to the first injection (Figure 5F). Similarly, the *t* = 2 h ratio of endoxifen-glucuronide to its parent molecule endoxifen showed a 3-fold increase compared to the first injection (Figure 5E). On the other hand, no difference was observed for 4OH-tamoxifen/tamoxifen (Figure 5A), N-desmethyltamoxifen/tamoxifen (Figure 5B), and endoxifen/4OH-tamoxifen ratios (Figure 5D). Together, these results indicate that tamoxifen metabolism is slightly potentiated following two subsequent injections of the drug.

As morphine has a short half-life in mice (30 min), we have performed three injections of morphine to reach adequate concentrations in the blood (Supplementary Figure 1). The highest concentrations of morphine and M3G in the blood were reached after 2 h (1,599 ± 336 pmol/ml and 9,773 ± 1,274 pmol/ml, respectively). Morphine was still present after 8 h, allowing a long-lasting competition with tamoxifen metabolism.

**Figure 6:**
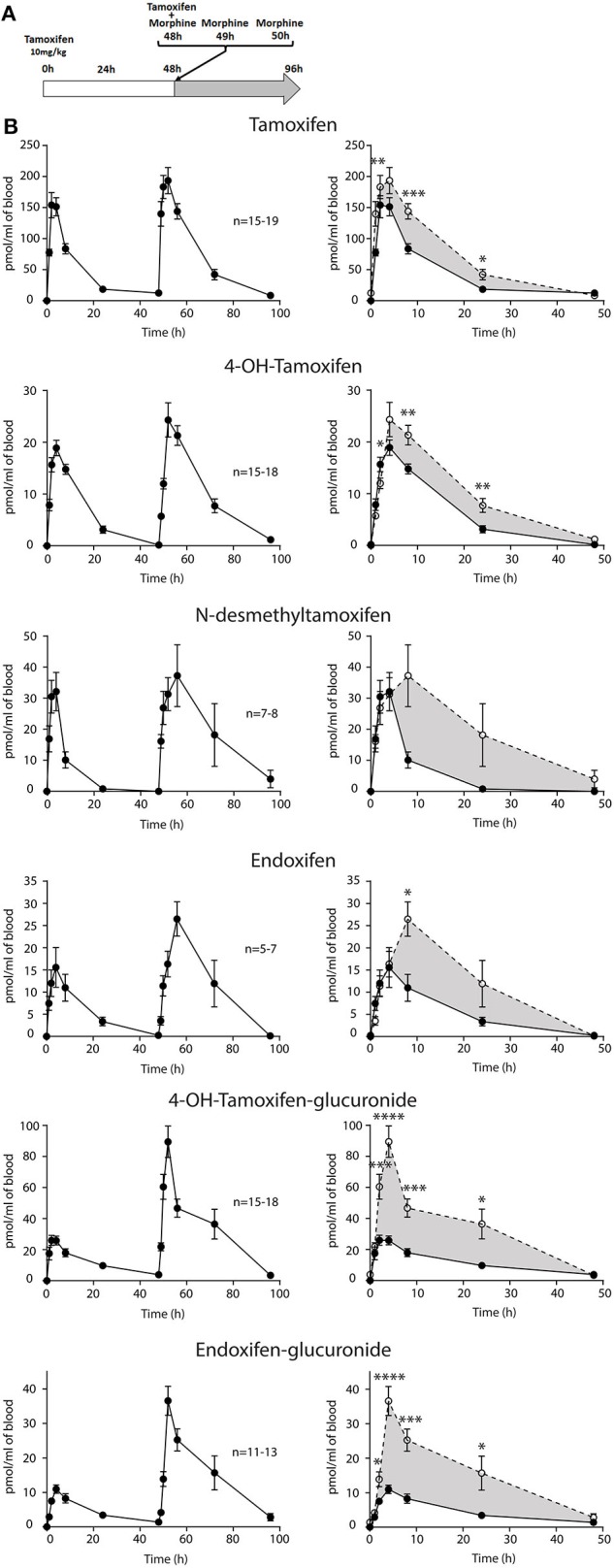
Morphine increases tamoxifen metabolism. Effect of three injections of morphine (10 mg/kg i.p.) on the blood concentrations of tamoxifen, 4OH-tamoxifen, N-desmethyltamoxifen, endoxifen, 4OH-tamoxifen-glucuronide, and endoxifen-glucuronide. **(A)** Protocol. Injections of NaCl 0.9% at 0, 1, and 2 h are not represented. **(B)** Left panels, levels of tamoxifen, 4OH-tamoxifen, N-desmethyltamoxifen, endoxifen, 4OH-tamoxifen-glucuronide, and endoxifen-glucuronide during 96 h. Right panels correspond to the superimposition of the first 0–48 h (white area) and last 48–96 h (gray area). Gray area corresponds to an increase of the quantity of the corresponding molecule after the second injection (48–96 h). Values are means ± SEM. *p < 0.05; **p < 0.01; ***p < 0.001.

**Figure 7:**
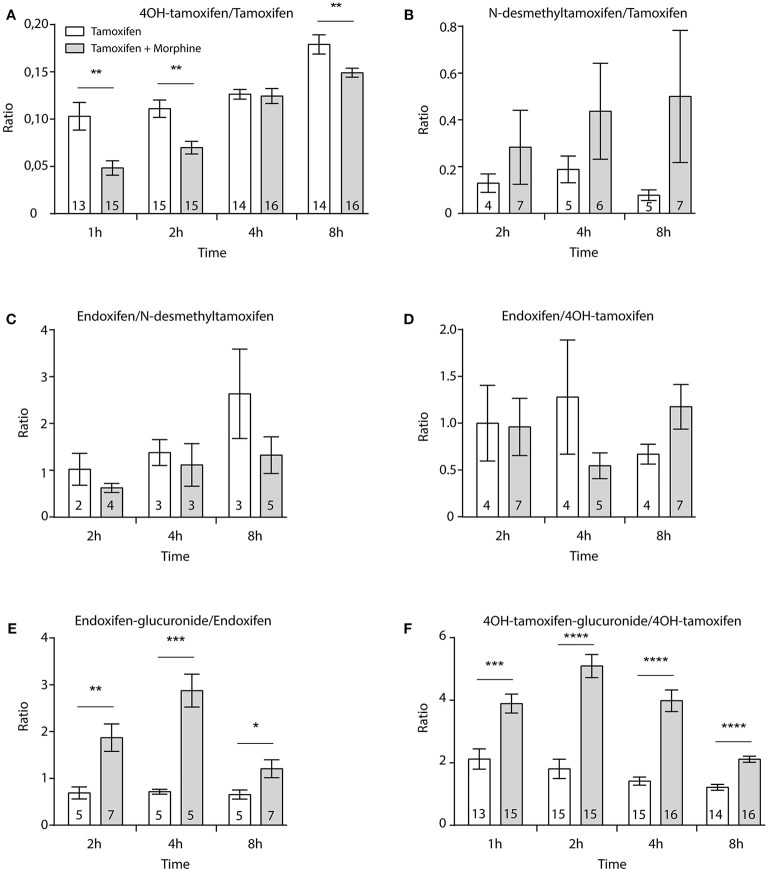
Morphine promotes the inactivation of tamoxifen through increased glucuronidation. Effect of three injections of morphine (10 mg/kg i.p.) on the ratio between metabolites and parent compounds. **(A)** 4OH-tamoxifen/tamoxifen, **(B)** N-desmethyltamoxifen/tamoxifen, **(C)** endoxifen/N-desmethyltamoxifen, **(D)** endoxifen/4OH-tamoxifen, **(E)** endoxifen-glucuronide/endoxifen, and **(F)** 4OH-tamoxifen-glucuronide/4OH-tamoxifen. *N* are indicated within columns. *t*-tests; *p < 0.05; **p < 0.01; ***p < 0.001; ****p < 0.0001.

Then, female mice were injected twice with tamoxifen (at 0 and 48 h) in addition to morphine (at 48, 49, and 50 h) and blood samples were collected (Figure 6A). Following morphine injections, the blood concentrations of tamoxifen, 4OH-tamoxifen, 4OH-tamoxifen-glucuronide, endoxifen, and endoxifen-glucuronide were significantly increased compared to the first injection of tamoxifen (Figure 6B). Only a tendency was observed for N-desmethytamoxifen. More importantly, ratios between 4OH-tamoxifen/tamoxifen (Figure 7A) were significantly decreased by 1/2- to 1/5-fold 1, 2, and 8 h after the injection of morphine, suggesting that 4OH-tamoxifen was processed into its metabolites at a faster rate in the presence of morphine. Indeed, the ratios of 4OH-tamoxifen-glucuronide/4OH-tamoxifen showed a significant increase (2- to 3-fold) at every time point (Figure 7F). Similarly, endoxifen-glucuronide/endoxifen ratios (Figure 7E) were dramatically increased (1.5- to 4-fold) at 2, 4, and 8 h after the injection of morphine. On the other hand, the ratios of N-desmethyltamoxifen/tamoxifen (Figure 7B), endoxifen/N-desmethyltamoxifen (Figure 7C) and endoxifen/4OH-tamoxifen (Figure 7D) were not altered by morphine administration. Together, these results indicate that the inactivation of tamoxifen and its active metabolites is exacerbated in the presence of equimolar amounts of morphine.

## Discussion

### Repeated Tamoxifen Treatment Potentiates Glucuronide Formation *in vivo*

Our results show that the blood formation pattern of N-desmethyltamoxifen and endoxifen is slightly modified *in vivo* after two subsequent tamoxifen treatments. Indeed, we observed a higher peak concentration in the case of N-desmethyltamoxifen and a slower elimination for endoxifen upon the second administration of tamoxifen. Furthermore, analysis of metabolic ratios revealed an increase in 4OH-tamoxifen-glucuronide and endoxifen-glucuronide formation compared to their parent drugs when animals received a second injection of tamoxifen. Such an increase of glucuronidation can be related to induction of the expression of UGTs present in the liver occurring 48 h after the first injection of tamoxifen. Indeed, it has been described that several xenobiotics are able to promote UGT expression by acting on regulatory elements in the cell (20). Tamoxifen acts as a selective modulator on the ER, which, in turn, modulates the activity of numerous transcription factors implicated in the regulation of gene expression. Importantly, tamoxifen has been shown to increase the expression of CYP enzymes involved in its own metabolism, such as CYP3A4 (21). In the same manner, one may hypothesize that the first injection of tamoxifen induced the expression of UGTs, resulting in a potentiation of 4OH-tamoxifen and endoxifen glucuronidation upon the second treatment.

Surprisingly, despite an increase in tamoxifen glucuronidation, we observed no concurrent decrease in the concentrations of 4OH-tamoxifen or endoxifen. The main degradation pathway of tamoxifen is glucuronidation, but significant amounts of its two active metabolites are eliminated through sulfation. Several sulfotransferase (SULT) isoforms (1A1, 1E1, and 2A1) have been implicated in the degradation of 4OH-tamoxifen (22). In addition, it has been shown *in vitro* that tamoxifen metabolites are able to inhibit SULT2A1 through mixed or non-competitive inhibition (23). Therefore, it is possible that our first tamoxifen administration inhibited SULT expression toward 4OH-tamoxifen and endoxifen. Thus, the balance between glucuronidation and sulfation could be modified without affecting 4OH-tamoxifen or endoxifen levels. Nevertheless, this hypothesis remains to be tested.

### Morphine Increases Glucuronidation of Tamoxifen Active Metabolites

Morphine was expected to reduce the glucuronidation of tamoxifen active metabolites through direct competition on the UGT-binding site as observed *in vitro*. Surprisingly, our results showed a dramatic increase in the levels of all active and inactive metabolites of tamoxifen when morphine was co-administered. The significant elevated levels of tamoxifen found in the blood after the coinjection with morphine may explain the increase observed for all compounds. This increase is likely to rely on differences of absorption due to drug–drug interactions with morphine rather than variability in tamoxifen injections. This point is strengthened by the fact that 19 mice were injected using a calibrated Hamilton syringe. Ratio between metabolites and their corresponding parent molecules were established to normalize the metabolite production with the tamoxifen injections. Analysis of the ratio revealed that morphine dramatically decreased the amount of 4OH-tamoxifen relative to that of its prodrug in the blood of tamoxifen-treated mice. This decrease is likely related to the concurrent massive increase of the glucuronidation of 4OH-tamoxifen and endoxifen.

It seems improbable that morphine would act as a cofactor of UGTs, allowing faster glucuronidation since it did not occur in our *in vitro* experiments. A potential impact of morphine on the entry of tamoxifen in hepatocytes is also unlikely because tamoxifen is known to cross the cell membrane passively (7), whereas morphine influx relies on transporters including organic cation transporter 1 (OCT1) (24). The last type of common molecular targets in the metabolism of tamoxifen and morphine are MRP (multidrug resistance-associated protein) and MDR (multidrug resistant protein) transporters driving M3G, 4OH-tamoxifen, endoxifen, 4OH-tamoxifen-glucuronide, and endoxifen-glucuronide out of the cell (7, 16, 25, 26). One hypothesis involving those transporters may be that morphine decreases the efflux rate of tamoxifen active metabolites (and thus increases their glucuronidation rate). Additional studies are needed to decipher the molecular mechanism underlying this atypical change in tamoxifen metabolism.

In conclusion, co-administration of morphine in mice appears to promote the inactivation of the potent 4OH-tamoxifen and endoxifen metabolites. In light of these findings, we hypothesize that morphine could reduce the potency of tamoxifen anticancer treatment in mice. Further studies should determine if the impact of morphine on tamoxifen metabolism is sufficient to result in changes in anticancer activity at therapeutic doses.

### Strengths and Limitations

We chose to associate morphine with tamoxifen to develop our methodology as it was expected to be a simple model focusing primarily on the glucuronidation process. Morphine is mainly metabolized by UGTs and was not expected to impact CYP activity. Morphine and tamoxifen co-treatments are given after surgeries or in the case of severe cancer pain (27). Otherwise, codeine and/or paracetamol are widely prescribed (8). In human, these two compounds are metabolized by the same CYPs (6D6/3A4) and UGTs (1A10, 1A4, 1A8, 2B7, and 2B15) (28, 29) as tamoxifen and might have a more complex impact on tamoxifen metabolic pathways (30, 31).

A main limitation of our study is that tamoxifen and morphine metabolisms differ in mice compared to humans. 4OH-tamoxifen is the major active mouse metabolite whereas endoxifen is found at greater concentrations in human serum. However, our approach using the isotopic dilution allowed us to observe non-negligible levels of both endoxifen and endoxifen-glucuronide in the blood of tamoxifen-treated mice. In mice, the Cyp2d gene cluster displays nine functional genes (Cyp2d9, Cyp2d10, Cyp2d11, Cyp2d12, Cyp2d13, Cyp2d22, Cyp2d26, Cyp2d34, and Cyp2d40), whereas humans only have one (CYP2D6) (4). Therefore, the presence of endoxifen suggests that CYP2D6 activity is rescued by an alternative CYP.

In addition, morphine is only metabolized into M3G in mice vs. M3G and M6G in humans (32, 33). Nevertheless, both species eliminate tamoxifen and morphine predominantly through glucuronidation. UGT2B7 (15), the main UGT involved in morphine metabolism in humans, is absent in mice. However, morphine and tamoxifen glucuronidation could be compensated by other enzymes including the mouse homologs of human UGT2B6, 2C9, 2C19, 3A4/5 (34), UGT2B36, and UGT2B21 (14, 15). These differences lead to a tamoxifen half-life of 27 h in humans and 6.8 h in mice (4), as well as a morphine half-life of 30 min in mice and 2 h in humans (32, 33). Despite the existence of mouse equivalents to human CYP and UGT isoforms, major differences in isoform sequence and expression patterns limit the extrapolation of mouse data to humans. The development of humanized mouse models for CYP and UGT genes will allow overcoming such issues (34, 35).

Drug–drug interactions can lead to severe adverse effects and predicting these interactions *in vivo* is challenging. Thus, the Food and Drug Administration (FDA) and European Medicines Agency (EMA) are frequently publishing new guidelines regarding *in vitro* and *in vivo* drug–drug interaction studies (36). We have used an *in vivo* methodology to monitor modulations of tamoxifen metabolism. Intraperitoneal injections of tamoxifen were used instead of oral administration (the typical route of administration in humans) in order to better control the given amounts of tamoxifen and morphine (37). Indeed, the most used method is intraperitoneal injection, because the amount of administered compound can be better controlled, but delivery by oral gavage is also possible. However, oral administration suffers from significant first-pass metabolism (38), which limits absorption (39) and introduces inter-individual variability in drug metabolism (40). The pharmacokinetics of tamoxifen were obtained by quantification of tamoxifen and its metabolites following an initial injection (10 mg/kg). Then, a second injection was used to determine its pharmacokinetics in the absence or the presence of the competing drug morphine. Therefore, it was possible to accurately compare tamoxifen pharmacokinetics in the same animal to assess its potential interaction with morphine *in vivo*. It is however important to determine whether an injection of the drug of interest can induce adaptive processes responsible for differences in its metabolism following a second injection or chronic treatment.

## Conclusions

In this study, we have investigated the effects of morphine on tamoxifen metabolism *in vitro* and *in vivo*. We have shown that *in vitro* morphine inhibits 4OH-tamoxifen glucuronidation. Conversely, morphine reduced the blood levels of 4OH-tamoxifen in mice, while the inactivation of tamoxifen active compounds through glucuronidation greatly increased.

Our results suggest that morphine co-treatment could dramatically affect tamoxifen efficacy and emphasize the need to test more common analgesics (e.g., codeine or paracetamol) in humans to re-evaluate the impact of pain treatments on anti-cancer drug metabolism and pharmacological activity.

## Data Availability Statement

The datasets generated for this study are available on request to the corresponding author.

## Ethics Statement

All animal procedures were performed in accordance with European directives (2010/63/EU) and were approved by the regional ethics committee and the French Ministry of Agriculture (license No. APAFIS#16827-2018092113192911 v4 to YG).

## Consent for Publication

All authors have approved the manuscript for submission.

## Author Contributions

Conceptualization: FG, VC, IW, and YG. Methodology: FG, VC, IW, PD, and YG. Investigation: FG, VC, IW, VH, TM, and A-SA. Writing—original draft: YG, FG, and PD. Writing—review and editing: YG, FG, A-SA, VH, TM, and PD. Funding acquisition, resources, and supervision: YG.

### Conflict of Interest

The authors declare that the research was conducted in the absence of any commercial or financial relationships that could be construed as a potential conflict of interest.

## Abbreviations

ACN: acetonitrile
ADME: absorption, distribution, metabolism, and/or excretion
AF: formic acid
AI: aromatase-inhibitors
CYP: cytochrome P450
ER: estrogen receptor
i.p.: injected intraperitoneally
IS: internal standard
LC-MS/MS: liquid chromatography-mass spectrometry/mass spectrometry
M3G: morphine-3-glucuronide
M6G: morphine-6-glucuronide
MOR: Mu opioid receptors
SEM: standard error of the mean
SERM: selective estrogen receptor modulator
TDM: therapeutic drug monitoring
UGT: UDP-glucuronosyltransferases
SULT: sulfotransferase.
