# Supplementary material for: Unveiling the Impact of Morphine on Tamoxifen Metabolism in Mice in vivo
Source: Front Oncol. 2020 Feb 21;10:25. doi: 10.3389/fonc.2020.00025 (PMC7046683; doi:10.3389/fonc.2020.00025)
Supplement: Supplementary file 1 [file Data_Sheet_1.pdf]

Supplementary Material

Supplementary Table 1- LC and MS/MS conditions for the purification, detection and quantification of tamoxifen, 4OH-tamoxifen, 4OH-tamoxifen-glucuronide, endoxifen, endoxifen-glucuronide, N-desmethyltamoxifen, morphine, M3G and their respective heavy tagged counterparts**.** Buffer A corresponded to ACN 1% / H_2O 98.9% / AF 0.1% (v/v/v), whereas buffer B was ACN 99.9 % / AF 0.1% (v/v).

HPLC gradient

**Time (min)**	0	2.5	4.5	8	12	15	15.5	22
**% B buffer**	0	0	40	60	98	98	0	0

MS ionization, selection, fragmentation and identification parameters

**Compound**	**Polarity**	**Precursor (m/z)**	**Product (m/z)**	Collision **Energy (V)**	**Ion product type**
**Tamoxifen**	Positive	372.23	72.26<br>129.11<br>327.10	24	Qualification & Quantification
**D5-Tamoxifen**	Positive	377.23	72.29<br>332.15<br>212.07	26	Qualification & Quantification
**4OH-tamoxifen**	Positive	388.17	72.33<br>193.92<br>223.03	21	Qualification &

					Quantification
**D5-4OH-tamoxifen**	Positive	393.25	72.26<br>227.92<br>166.10	21	Qualification<br>&<br>Quantification
**4OH-tamoxifen-glucuronide**	Positive	564.2	283.294<br>388.15<br>484.30	13<br>24<br>14	Qualification<br>&<br>Quantification
**D5-4OH-tamoxifen-glucuronide**	Positive	569.34	393.22	23	Qualification<br>&<br>Quantification
**Endoxifen**	Positive	374.21	58.40<br>152.13<br>223.04	20.16<br>18.75<br>18.80	Qualification<br>&<br>Quantification
**Endoxifen-glucuronide**	Positive	550.18	58.373<br>223.04<br>374.16	32<br>30<br>22	Qualification<br>&<br>Quantification
**D5-Endoxifen-glucuronide**	Positive	555.18	379.16<br>228.04<br>63.37	22	Quantification<br>&<br>Qualification
**N-desmethyltamoxifen**	Positive	358.20	58.40<br>129.11<br>206.89	20<br>26<br>19	Quantification<br>&<br>Qualification

**D5-N-desmethyltamoxifen**	Positive	363.20	58.39<br>134.03<br>212.06	20<br>28<br>19	Quantification<br>&<br>Qualification

Limits of detection and quantification

	LOD (pmol)	LOQ (pmol)
**Tamoxifen**	0,019	0,038
**4-hydroxytamoxifen**	0,078	0,156
**Endoxifen**	0,078	0,156
**N-desmethyl-tamoxifen**	0,078	0,156
**4-OH-O-Glucuronide**	0,078	0,156

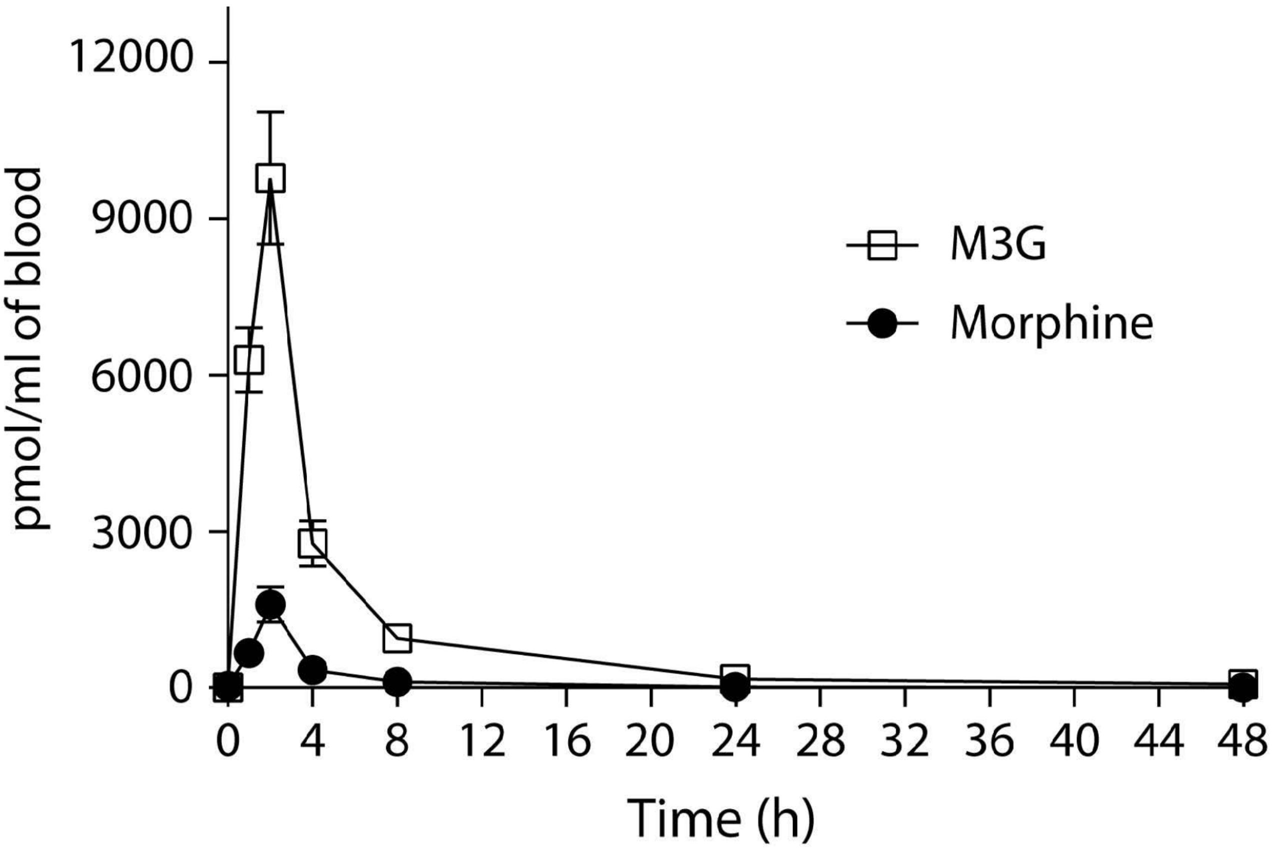

Supplementary Figure 1. Morphine and M3G blood levels. N=13, values are means ± SEM
